# Cutaneous diffuse large B‐cell lymphoma

**DOI:** 10.1002/ccr3.1283

**Published:** 2017-11-28

**Authors:** Filipa Duarte Ribeiro, Henrique Coelho, David Tente, Margarida Badior

**Affiliations:** ^1^ Internal Medicine Department Centro Hospitalar Vila Nova de Gaia/Espinho Rua Conceição Fernandes 4434‐502 Vila Nova de Gaia Portugal; ^2^ Haematology Department Centro Hospitalar Vila Nova de Gaia/Espinho Rua Conceição Fernandes 4434‐502 Vila Nova de Gaia Portugal; ^3^ Pathology Department Centro Hospitalar de Vila Nova de Gaia/Espinho Rua Conceição Fernandes 4434‐502 Vila Nova de Gaia Portugal

**Keywords:** B cells, chemotherapy, cutaneous lesion, Non‐Hodgkin lymphoma

## Abstract

Cutaneous diffuse large B‐cell lymphoma accounts for ~6% of all cutaneous lymphomas. It is associated with poor prognosis, and solitary lesions are relatively rare. It often requires an aggressive approach with multi‐agent chemotherapy and radiotherapy. It is important to recognize these cases in order to offer rapid and appropriate management.

A previously healthy 25‐year‐old man presented to the Emergency Department with a painful, ulcerated swelling lesion on the scalp (Fig. 1A) that was growing over the last 6 months. A biopsy was performed, and the anatomopathological study favored the diagnosis of a cutaneous diffuse large B‐cell lymphoma, leg type (Fig. 1B: diffuse lymphoid dermal and hypodermic infiltration of predominantly large centroblastic B cells; HE, x20 and Fig. 1C: mixed medium to large cells, predominantly centroblastic; HE, x400). The neoplastic B‐cell phenotype was CD20^+^, CD79a^+^, CD5^−^, CD30^−^, CD10^−^, BCL6^+^, MUM1^−^, BCL2^+^, KI67 index >90%, CD21^−^. Laboratory studies did not show abnormalities, and viral markers were negative. The brain computed tomography (CT) scan showed a soft tissue tumor on the right parietal region (63 × 33 mm), with no evidence of bone invasion (Fig. 1D). Staging with bone marrow biopsy, total body CT, and lumbar puncture showed a stage T1N0M0. Treatment according to the R‐CHOP (rituximab, cyclophosphamide, doxorubicin, vincristine, and prednisolone) protocol was started with almost complete resolution of the lesion after 2 cycles (Fig. 1E). He was proposed to consolidation with radiotherapy after six cycles of chemotherapy 1, 2.

**Figure 1:**
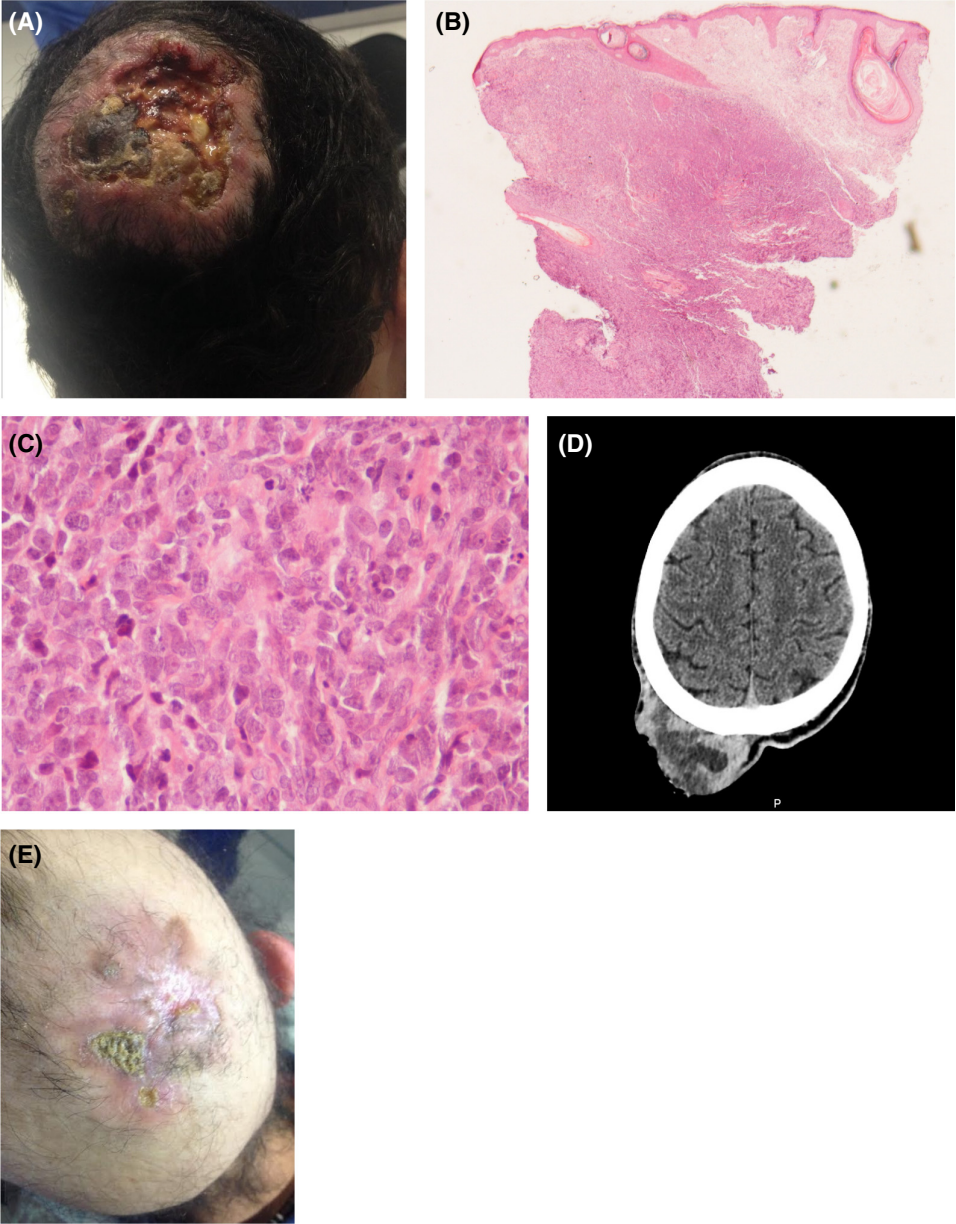
(A) ‐ swelling lesion on the scalp. (B) ‐ diffuse lymphoid dermal and hypodermic infiltration of predominantly large centroblastic B cells (HE, x20). (C) ‐ mixed medium to large cells, predominantly centroblastic (HE, ×400). (D) ‐ brain computed tomography showing a soft tissue tumor on the right parietal region (63x33mm), with no evidence of bone invasion. (E) ‐ scalp lesion after 2 cycles of chemotherapy.

## Authorship

FDR: involved in the conception, acquisition, analysis and interpretation of data, drafted the article or revised it critically for important intellectual content, provided agreement to be accountable for the article and gave final approval of the version to be submitted and the revised version. HC: involved in the conception, acquisition, analysis and interpretation of data, revised the article critically for important intellectual content, provided agreement to be accountable for the article and gave final approval of the version to be submitted and the revised version. DT: collected pathology images, involved in the acquisition, analysis and interpretation of data, provided agreement to be accountable for the article and give final approval of the version to be submitted and the revised version. MB: involved in the conception, acquisition, analysis and interpretation of data, revised the article critically for important intellectual content, provided agreement to be accountable for the article and give final approval of the version to be submitted and the revised version.

## Conflict of Interest

None declared.
